# Inhibitory Infrared Light Attenuates Mitochondrial Hyperactivity and Accelerates Restoration of Mitochondrial Homeostasis in an Oxygen–Glucose Deprivation/Reoxygenation Model

**DOI:** 10.3390/antiox14091119

**Published:** 2025-09-15

**Authors:** Lucynda Pham, Tasnim Arroum, Paul T. Morse, Jamie Bell, Moh H. Malek, Thomas H. Sanderson, Maik Hüttemann

**Affiliations:** 1 Center for Molecular Medicine and Genetics, Wayne State University, Detroit, MI 48201, USA; lucynda.pham@med.wayne.edu (L.P.); ho0066@wayne.edu (T.A.); morsepa@wayne.edu (P.T.M.); jbell@dmc.org (J.B.); 2 Division of Pediatric Critical Care, Children’s Hospital of Michigan, Central Michigan University, Detroit, MI 48201, USA; 3 Department of Health Care Sciences, Eugene Applebaum College of Pharmacy & Health Sciences, Wayne State University, Detroit, MI 48201, USA; en7488@wayne.edu; 4 Department of Emergency Medicine, University of Michigan Medical School, Ann Arbor, MI 48109, USA; thsand@med.umich.edu; 5 Department of Biochemistry, Microbiology, and Immunology, Wayne State University, Detroit, MI 48201, USA

**Keywords:** mitochondria, ischemia/reperfusion injury, cytochrome *c* oxidase, mitochondrial membrane potential, reactive oxygen species, near-infrared light, phosphorylations

## Abstract

Ischemia/reperfusion (I/R) injury following stroke results in increased neuronal cell death due to mitochondrial hyperactivity. Ischemia results in loss of regulatory phosphorylations on cytochrome *c* oxidase (COX) and cytochrome *c* of the electron transport chain (ETC), priming COX for hyperactivity. During reperfusion, the ETC operates at maximal speed, resulting in hyperpolarization of the mitochondrial membrane potential (ΔΨ_m_) and reactive oxygen species (ROS) production. We have shown that COX-inhibitory near-infrared light (IRL) provides neuroprotection in small and large animal models of brain I/R injury. IRL therapy is non-invasive and non-pharmacological and does not rely on blood flow. We identified specific wavelengths of IRL, 750 and 950 nm, that inhibit COX activity. To model the mitochondrial effects following neuronal I/R, SH-SY5Y cells underwent oxygen–glucose deprivation/reoxygenation (OGD/R) ± IRL applied at the time of reoxygenation. Untreated cells exhibited ΔΨ_m_ hyperpolarization, whereas IRL treated cells showed no significant difference compared to control. IRL treatment suppressed ROS production, decreased the level of cell death, and reduced the time to normalize mitochondrial activity to baseline levels from 4–5 to 2.5 h of reperfusion time. We show that IRL treatment is protective by limiting ΔΨ_m_ hyperpolarization and ROS production, and by speeding up cellular recovery.

## 1. Introduction

Brain ischemia, caused by thrombotic occlusion locally or cardiac arrest globally, is a leading cause of death and disability, with about 115,560 ischemic strokes and 417,957 cardiac arrests in the US annually [1]. Prompt restoration of blood flow, or reperfusion, to the ischemic tissue is the only method to reduce neurological damage through ischemic necrotic cell death. However, reperfusion generates additional tissue damage and is a primary cause of poor clinical outcomes and is referred to as reperfusion injury. The ischemic core that relies exclusively on blood flow from the obstructed vessel is surrounded by tissue, which is of clinical interest as a fraction of the neurons here die later following reperfusion and may be salvageable. This area is referred to as the penumbra, which is not fully reliant on the obstructed vessel because it is partially perfused by collateral blood flow. The neurons in the penumbra are subjected to further damage caused by reperfusion injury due to mitochondrial hyperactivation and ROS production and are the primary therapeutic target of interest [2].

Mitochondria are the primary source of reactive oxygen species (ROS) production via the electron transport chain (ETC) complexes I (NADH dehydrogenase) and III (*bc*1 complex) [3]. The coupling of electron transfer to proton pumping from the matrix into the intermembrane space (IMS) generates the mitochondrial membrane potential (ΔΨ_m_). During ischemia, where blood flow and therefore oxygen supply to the tissue is compromised, the ETC comes to a halt as oxygen, the terminal electron acceptor and substrate of COX, is absent and ATP is rapidly depleted. ROS production and ΔΨ_m_ is correlated; when ΔΨ_m_ exceeds the optimal intermediate range, there is an exponential increase in ROS [4,5,6,7,8]. Under physiological conditions, this process is tightly regulated to prevent excessive and harmful ROS while optimizing ATP production [9]. The reaction between cytochrome *c* (Cyt*c*) and cytochrome *c* oxidase (COX) is the proposed rate-limiting step, which has a major influence on regulating the ΔΨ_m_ [10,11,12,13,14,15]. Therefore, both Cyt*c* and COX are under tight regulatory control, which includes allosteric regulation by ATP/ADP and other small molecules, tissue-specific isoforms, and post-translational modifications (PTMs), including phosphorylations [16]. Utilizing methods of mitochondrial and COX/Cyt*c* isolations that preserve phosphorylations, it has been shown that both proteins are reversibly phosphorylated under physiological conditions. These phosphorylated sites alter electron transfer kinetics that lead to partial inhibition and, under normal conditions, healthy cellular respiration [17,18,19].

We and others have demonstrated that following cellular stress, such as ischemia, the phosphorylation state of Cyt*c* and COX is altered, changing their regulation and thus activity [20,21]. During ischemia, there is an influx of cytosolic calcium [Ca^2+^], which is sequestered by mitochondria [22,23,24]. It has been postulated that the purpose of increased mitochondrial Ca^2+^ is to stimulate mitochondrial respiration as an attempt to increase ATP production [9,25]. Additionally, Ca^2+^ and mitochondrial Ca^2+^ overload have been shown to increase respiration and excessive ROS generation [26]. Calcium can activate downstream stress signaling pathways and mitochondrial phosphatases, such as the calcium-dependent Ser/Thr phosphatase calcineurin [27]. In support of this concept, it has been shown that during ischemia and Ca^2+^ accumulation, there is an overall dephosphorylation of mitochondrial proteins, including COX [28,29,30]. The dephosphorylation of COX alters enzyme kinetics in addition to the loss of allosteric inhibition by ATP. We have previously shown that under ischemia, neuronal Cyt*c* is dephosphorylated on residue Ser47, resulting in increased mitochondrial activity and ROS production [31]. Therefore, ischemia attempts to increase energy production and thus respiration, which is futile due to the absence of oxygen and results in a mitochondrial state that is primed for hyperactivity when reperfusion takes place [32,33]. When oxygen returns, the dephosphorylation state of Cyt*c* and COX promotes rapid restoration of ΔΨ_m_ and ATP. However, due to the loss of regulatory PTMs and the ATP allosteric inhibition, ΔΨ_m_ surpasses physiological levels, resulting in ΔΨ_m_ hyperpolarization. This results in a “ROS burst”, which causes extensive damage to cellular components and initiates apoptosis [34,35]. In support of the central role of mitochondria during I/R injury, it has been shown that the majority of ROS generated during global brain ischemia occurs within the first 15 min of reperfusion [36], but ΔΨ_m_ hyperpolarization remains high for an extended time after this initial phase, as was shown in a two-photon mouse brain imaging study following global brain I/R [37].

There have been extensive efforts to target and prevent mitochondrial hyperactivity to mitigate I/R injury by targeting the ETC complexes [38,39]. For instance, inhibiting complex I has been shown to prevent ΔΨ_m_ hyperpolarization and subsequently control ROS levels [40]. Additionally, direct ROS targeting by endogenous antioxidants in transgenic animals has shown neuroprotection, highlighting the role mitochondrial ROS play in I/R injury [41,42,43,44]. However, pharmacological approaches have proven ineffective, likely due to their reliance on blood flow to reach the target tissue, which is compromised during ischemia, leading to a delay in reaching the target tissue and in building up effective concentrations. Additionally, the clinical efficacy of ROS scavengers has been inconclusive due to their short half-lives and narrow therapeutic windows [45,46,47].

Near-infrared light (IRL) is known to be primarily absorbed by COX due to its two copper centers that are crucial for its enzymatic activity [48,49,50,51]. It was initially believed that IRL consistently activated COX activity, which is true for certain wavelengths [52,53,54]. However, we have previously identified and characterized specific wavelengths of IRL (750 and 950 nm) that inhibit COX enzymatic activity [55,56,57]. We have shown that these inhibitory wavelengths provide robust neuroprotection in brain I/R injury animal models, including both rodent and clinically relevant pig models. We propose that by mildly inhibiting COX activity at the start of reperfusion, it is possible to prevent ΔΨ_m_ hyperpolarization and therefore *prevent* ROS generation. This approach is markedly different from pharmacological approaches that attempt to scavenge already generated ROS.

We here studied inhibitory IRL protection against I/R injury in an oxygen–glucose deprivation/reoxygenation (OGD/R) cell culture model using the human neuroblastoma SH-SY5Y cell line. COX activity was partially inhibited by IRL at the onset of reperfusion, preventing ΔΨ_m_ hyperpolarization and consequently reducing ROS production. SH-SY5Y cells are a commonly used cell culture model to study mechanisms related to stroke and I/R injury, particularly by performing OGD/R. We show that inhibitory IRL wavelengths lower ΔΨ_m_ to control levels following early reoxygenation, which prevents mitochondrial superoxide generation. Additionally, 2 h IRL treatment protects against apoptosis in late-stage reperfusion. We also show by activity measurements for cells that underwent OGD/R, COX activity was increased, likely due to dephosphorylation-mediated activation, which subsequently returned to control levels after two hours of IRL treatment during reperfusion. Our work adds to the body of work highlighting the clinical potential of inhibitory IRL as a therapeutic strategy for preventing neuronal damage associated with I/R injury. Building on our previous work that has shown protection in several animal models, we now provide additional mechanistic insights into the therapeutic effects using an established cell culture model.

## 2. Materials and Methods

### 2.1. Cell Culture

SH-SY5Y human neuroblastoma cells from American Tissue Culture Collection (CRL-226 ATCC, Manassas, VA, USA) were cultured in Dulbecco’s modified Eagle’s medium (DMEM) (#11965-092 Gibco, Grand Island, NY, USA) supplemented with 10% fetal bovine serum (FBS) and 1 mg/mL penicillin/streptomycin (#15140122 Gibco). Cells were maintained at 37 °C with 5% CO_2_.

### 2.2. Oxygen–Glucose Deprivation/Reoxygenation (OGD/R) and IRL Treatment

**Figure 1:**
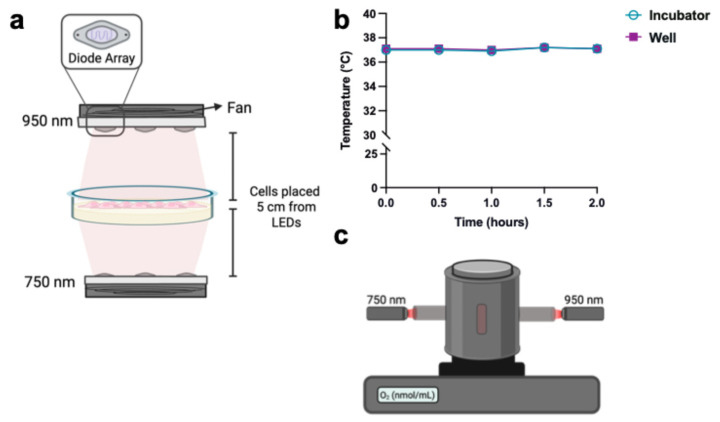
Schematic for treatment of cells with IRL. (**a**) The cell culture incubator was equipped with a cooling coil (not shown) set to remove any additional heat generated by the LEDs. Adherent cells are placed 5 cm from each LED to achieve the desired power density. The 750 nm is below the cells and irradiates from the bottom of the dish. The 950 nm is on the top of the cells and irradiates from above. The LEDs contain a fan to draw excess heat away from the cells by directing airflow away from the cells. (**b**) Measured temperature using thermocouples placed inside the wells containing media over 2 h with IRL on in the chamber. (**c**) Schematic of the custom light-protected Clark-type oxygen electrode chamber attached to a light-guide connecting the LEDs with the oxygen electrode chamber to illuminate the oxygen consumption rate measurement chamber with IRL wavelengths.

To induce ischemia/reperfusion injury in the SH-SY5Y cells, oxygen–glucose deprivation/reoxygenation (OGD/R) was performed. The OGD media consisted of glucose-free DMEM (#A1443001, Gibco) without glutamine, FBS, or phenol red and was bubbled with nitrogen gas for 1 h prior to the experiment. The reperfusion media consisted of DMEM supplemented with 1 mM sodium pyruvate, 5 mM glucose, without FBS or phenol red. Cells were washed with phosphate-buffered saline (PBS), and the culture medium was exchanged to either OGD or reperfusion media for the controls. The control cells were maintained at atmospheric O_2_ and 5% CO_2,_ while OGD was performed for 90 min or 6 h at 1% O_2_ and 5% CO_2_. Following OGD, the media was exchanged to the reperfusion media and placed back in the incubator at atmospheric O_2_ and 5% CO_2_ for the indicated time points. Light-emitting diodes (LEDs) obtained from Roithner Lasertechnik (Vienna, Austria). Six LED-array-60 chips were used for each wavelength (750 nm, LED750-66-60; 950 nm, LED950-66-60). Diodes were cooled using a fan (EC3010M05X; Evercool, New Taipei City, Taiwan) operated in reverse mode, directing the warm air away from the plate containing the cells. The diodes were calibrated using an optical power and energy meter (842-PE, Newport, Irvine, CA, USA). At the start of simulated reperfusion, the IRL treated cells were placed in a separate incubator with an LED array set-up and placed at a distance of 5 cm from the LEDs to achieve measured power outputs of 20 µW/cm^2^ for the 750 and 950 nm individually which was applied continuously for the indicated time points. See schematic (Figure 1a). As an additional control, temperature was measured using a 4-channel datalogging thermometer (Extech SDL200; Teledyne, Wilsonville, OR, USA) and remained constant for hours with the light on (Figure 1b). Of note, we measured the temperature in the well with cell culture medium, which remained at ~37 °C.

### 2.3. Mitochondrial Membrane Potential (ΔΨ_m_) and Superoxide Measurement

To measure relative changes in ΔΨ_m_, the ratio-metric probe JC10 (#ENZ-52305, Enzo Life Sciences, Farmingdale, NY, USA) was used following OGD. Briefly, cells underwent OGD as described above, and at the time of reperfusion, the media contained 10 µM of the probe. JC10 emits a green fluorescence as a monomer and, under increased ΔΨ_m_, accumulates in the mitochondria, forms aggregates, and emits a red fluorescence. Therefore, relative changes can be measured by quantifying the red/green fluorescence. Cells were seeded in black sided 96-well plates at 40,000 cells per well. Green (485/527 nm ex/em) and red (485/590 nm ex/em) fluorescence was measured using the BioTek Cytation C10 (BioTek Instruments Inc., Winooski, VT, USA) plate reader, and representative fluorescent images were taken after.

To measure relative superoxide levels, the fluorescent probe 500 nM MitoSOX Red (#M36008, Invitrogen; Carlsbad, CA, USA) was following OGD/R. For normalization purposes and to account for cell size and amount, 200 nM MitoView Green (#70054, Biotium, Freemont, CA, USA) was used to stain mitochondria. MitoSOX Red fluorescence was taken with a customized filter LED cube (396/610 nm ex/em; BioTek Instruments Inc.) to prevent detection of non-specific oxidized products and to minimize background.

### 2.4. Cytochrome c Oxidase Activity Measurement

Cytochrome *c* oxidase (COX) activity was measured using the Clark-type oxygen electrode (Oxygraph^+^ system, Hansatech, Pentney, Norfolk, UK). Cells were seeded in 6-well plates at 300,000 cells/well and, following OGD/R for the indicated time points, were washed with PBS and collected in 1.5 mL Eppendorf tubes. The cells were resuspended in COX solubilization buffer (10 mM K-HEPES, 40 mM KCl, 10 mM KF, 2 mM EGTA, 1% Tween-20, pH 7.4) and briefly sonicated on ice-slurry (3 secs, two pulses, energy setting 4; Sonic Dismembrator Model 100 Ultrasonicator, Fisher Scientific, Waltham, MA, USA). Importantly, 220 µL of each sample was transferred into the electrode chamber. Oxygen reduction rate in chamber was recorded at 25 °C and analyzed using the Oxytrace^+^ v.1.0.48 software (Hansatech). To calculate maximal COX activity, the basal oxygen reduction rate, prior to addition of cytochrome *c* and ascorbate, was subtracted from the rate measured after addition of sodium ascorbate (20 mM) and 30 µM cytochrome *c* (Sigma Aldrich, St. Louis, MO, USA, #C3131). The resulting value was normalized to protein concentration determined with the DC Protein Assay Kit (Bio-Rad #5000111, Hercules, CA, USA). Results are reported as nmol O_2_ reduced/min/µg protein.

### 2.5. Live Cell Oxygen Consumption Rate Measurements

For OCR measurements of live cells under IRL irradiation, a custom light-shielded Clark-type oxygen electrode chamber was used (Hansatech, Pentney, Norfolk, UK) (Figure 1c). The IRL of the LEDs was directed into the chamber and illuminated at a power density of 10 mW/cm^2^ (Figure 1c). For basal OCR measurements, cells (5 × 10^5^ cells per measurement) were left intact (not permeabilized or sonicated) and maintained in suspension in reperfusion media supplemented with glucose and sodium pyruvate as substrates to support the oxidative phosphorylation machinery. OCR was recorded using the Oxytrace^+^ v.1.0.48 software (Hansatech) in the presence or absence of IRL illumination, comparing control cells to those subjected to OGD/R.

### 2.6. Western Blotting

Total cell lysate was prepared by adding RIPA lysis buffer (50 mM Tris-HCl, pH 8.0, 150 mM NaCl, 1% NP-40, 0.5% sodium deoxycholate, 0.1% sodium dodecyl sulfate (SDS)) supplemented with protease inhibitor cocktail (PIC) (MilliporeSigma, #P8340, Burlington, MA, USA). The samples were sonicated and centrifuged at 10,000× *g* for 10 min at 4 °C, and protein concentration was determined using the DC protein assay kit (Bio-Rad). A total of 10 µg cell lysate was run on a 4–15% mini-protean TGX precast gel (Bio-Rad, #4561085). The gel was transferred onto a PVDF membrane (Bio-Rad, #1620177) by wet transfer at 10 V overnight at 4 °C. Blocking reagent and antibody dilutions were added to 5% bovine serum albumin (BSA) in 1× tris-buffered saline + 1% tween-20 (TBS-T). Membranes were incubated with primary antibodies for 1 h at room temperature or overnight at 4 °C. Antibodies were DRP1 (pS616) (1:1000, Thermo Fisher Scientific, #PA5-64821); DRP1 (1:1000, Cell Signaling Technology (CST), Danvers, MA, USA, #D6C7); VDAC (1:1000, CST, #4661S); 4-HNE, (1:1000, abcam, Cambridge, UK, #ab46545); β-actin (1:50,000, ProteinTech, Rosemont, IL, USA, #6008-1-Ig); cleaved caspase-3 (1:1000, CST, #9664S), pan caspase-3 (1:1000, CST, #14220S), PARP (1:1000, CST, #95425), TUBULIN (1:1000, Proteintech, #11224-1-AP). Secondary antibodies were incubated for 1 h at room temperature. Anti-Rabbit IgG HRP-linked (CST, #7074S) at 1:5000 and anti-mouse IgG HRP-linked (CST, #7076S) at 1:8000. Blot visualized using Pierce ECL Western blot substrate (Thermo Fisher Scientific, #32106) and imaged on a ChemiDoc MP imaging system (BioRad, #12003154).

### 2.7. Phosphorylated Protein Detection Using ProQ Diamond Phosphoprotein Stain

Cells that underwent OGD only (90 min) were collected for mitochondrial fractionation. Mitochondria were isolated by differential centrifugation. Briefly, cells were resuspended in hypotonic buffer (10 mM NaCl, 1.5 mM MgCl_2_, 10 mM Tris-HCl (pH 7.5)) for 10 min, followed by homogenization (80–120 strokes) using a Teflon homogenizer. Some of the sample was saved as total protein lysate, followed by the addition of mitochondria stabilization buffer (MSB; 210 mM mannitol, 70 mM sucrose, 5 mM Tris-HCl (pH 7.5), 1 mM EDTA (pH 7.5)) and centrifuging at 800× *g* for 5 min at 4 °C. The supernatant was collected and centrifuged at 10,000× *g* for 10 min at 4 °C. The final mitochondrial pellet was resuspended in MSB, and protein concentration was measured. To digest any cytosolic contamination and outer mitochondrial proteins, 100 µg mitochondria were taken and incubated with Proteinase K (Qiagen, Hilden, Germany, #1018332) for 30 min at room temperature on a shaker. PIC and phosphatase inhibitors (1 mM activated sodium vanadate and 10 mM KF) were added. Mitochondria were washed 3 times to remove proteinase K. RIPA and SDS loading buffer were added. The total lysate and mitochondrial fractions were run on a 4–15% TGX mini gel (Bio-Rad, #4561085). To stain for phosphorylated proteins, ProQ diamond phosphoprotein stain (Invitrogen, #P33300) was used according to manufacturer’s protocol. The gel was imaged using the ChemiDoc MP imaging system with ImageLab Touch software v.2.3.0.07 using the ProQ Diamond gel imaging function. To stain for total protein on the same gel, Sypro Ruby Protein gel stain (Invitrogen, #S12001) was used according to manufacturer’s protocol and imaged using the ChemiDoC MP Sypro Ruby gel imaging function.

### 2.8. Annexin V/Propidium Iodide Staining and Fluorescence-Activated Cell Sorting

Cell death was measured after annexin V and propidium iodide (PI) staining using fluorescence-activated cell sorting (FACS) analysis. Cells underwent OGD/R, where reoxygenation time was 18 h. Cells were harvested and resuspended in 1× annexin V binding buffer (FITC annexin V apoptosis kit I, BD Pharmigen, Franklin Lakes, NJ, USA, #556547, RRID: AB_2869082). A total of 1 × 10^6^ cells were stained. Data was collected through the Microscopy, Imaging & Cytometry Resources (MICR) facility at Wayne State University using the Cytek Northern Lights cytometer (Cytek Biosciences, Fremont, CA, USA). The data was analyzed using the FCS Express v7.0 software (De Novo Software, Glendale, CA, USA).

### 2.9. Statistical Analysis

Data are shown as means, and error bars represent standard deviation between independent wells or dishes. Statistical analysis of the data was performed using Graphpad Prism v10 (Graphpad Software, San Diego, CA, USA). For comparisons with only two groups, unpaired student’s two-tailed *t*-test was performed assuming equal variation was performed. For more than 2 groups, one-way ANOVA followed by Tukey’s post hoc test for multiple comparisons was performed. The *p*-values are indicated in the figure legends, where a *p*-value < 0.05 was considered statistically significant. To quantify Western blot and phosphoprotein staining images, Fiji ImageJ v.2.16.0/1.54p was used to perform densitometric analyses.

## 3. Results

### 3.1. Oxygen–Glucose Deprivation Leads to a Decrease in Mitochondrial Protein Phosphorylation and Increased COX Activity

**Figure 2:**
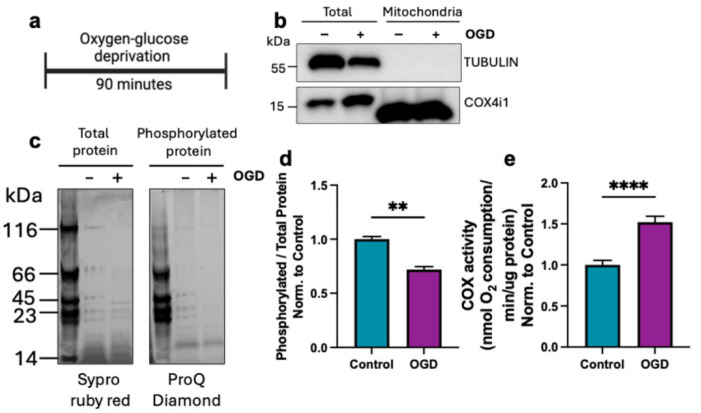
Oxygen–glucose deprivation decreases mitochondrial protein phosphorylation. (**a**) Cells underwent 90 min of oxygen–glucose deprivation (OGD; 1% O_2_) and were collected for mitochondrial isolation. (**b**) Western blot showing mitochondrial fractions using cytosolic marker (α-tubulin) and mitochondrial marker (COX4i1). (**c**) Phosphorylated mitochondrial protein gel staining (ProQ Diamond) followed by total protein staining (Sypro Ruby Red). (**d**) Quantification of phosphorylated protein signal normalized to total protein (*n* = 3). (**e**) COX activity measurements using sonicated cells in COX-solubilization buffer normalized to protein amount (*n* = 3). Bars represent means ± standard deviation and are presented as normalized to control. Statistical analysis was performed by unpaired Student’s t-test. Statistical significance was defined as *p* < 0.05, with ** indicating *p* < 0.01 and **** indicating *p* < 0.0001.

We have previously identified two wavelengths of IRL that partially inhibit COX activity. It has been known that mitochondrial hyperactivity occurs during early reperfusion and is the cause of ROS production, and that use of IRL at the time of reperfusion provides robust neuroprotection [55,56,57]. To further study how inhibitory IRL provides neuroprotection in live cells, we utilized the OGD/R cell culture model. Using SH-SY5Y cells, OGD with a 1% O_2_ atmosphere was performed for 90 min (Figure 2a). Cells were collected, and mitochondria were purified. Western blot analysis of cytosolic (Tubulin) and mitochondrial (COX4i1) markers were used to assess purity of the fractionation (Figure 2b). Mitochondrial fractions were run on an SDS-PAGE gel followed by staining with ProQ Diamond that specifically detects phosphorylated proteins [58] (Figure 2c). This was followed by Sypro Ruby Red staining to analyze total protein levels on the same gel. Relative quantification of phosphorylated mitochondrial proteins normalized to total protein showed that after 90 min of OGD, there was an overall decrease in phosphorylated mitochondrial proteins (Figure 2d). These results confirm previous findings of hypoxia-mediated dephosphorylation of mitochondrial proteins [28,31]. Concurrently, we observed a significant increase in oxygen consumption rate (OCR), a direct measurement of COX activity (Figure 2e). This observation is consistent with earlier reports of increased COX activity in brain tissue following ischemia, likely mediated by a loss of regulatory phosphorylations on COX [55].

### 3.2. Inhibitory Infrared Light Treatment Prevents ΔΨ_m_ Hyperpolarization and Suppresses Mitochondrial Superoxide Production During Early Reperfusion

**Figure 3:**
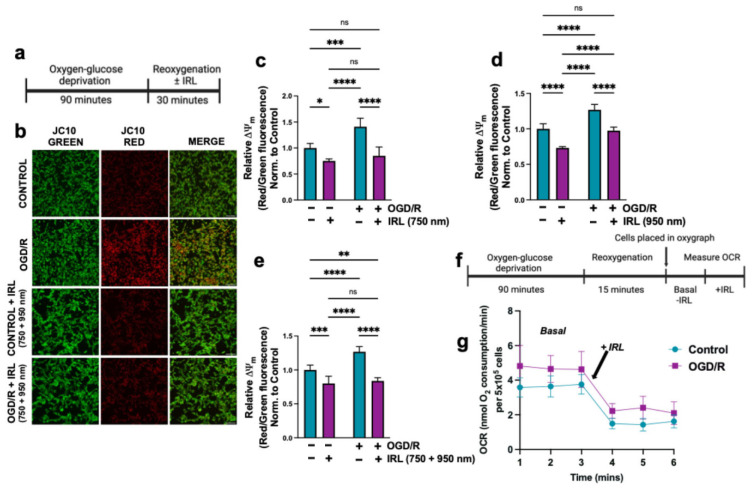

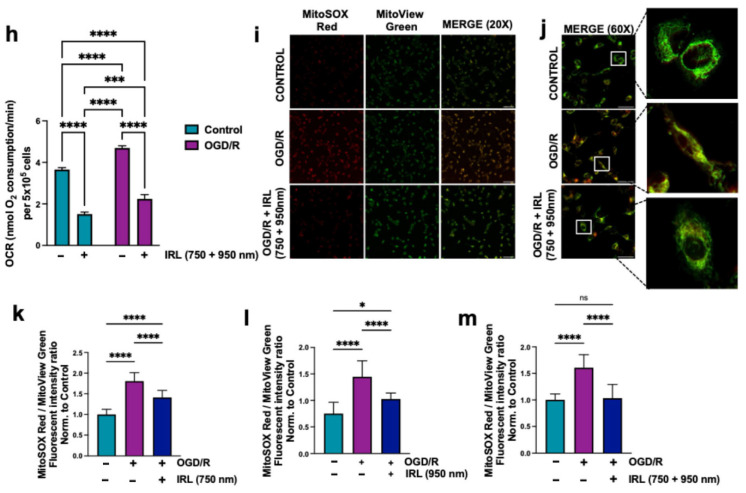
Mitochondrial ΔΨ_m_ hyperpolarization and increased superoxide during early reperfusion. (**a**) Cells underwent 90 min of OGD, followed by 30 min of reoxygenation with or without IRL (750, 950, or 750 + 950 nm). (**b**) Representative fluorescent images of the JC10 probe at 20X magnification. Scale bar, 100 µm. Relative changes in ΔΨ_m_ measured by JC10 red/green fluorescence for (**c**) 750 nm, (**d**) 950 nm, and (**e**) 750 + 950 nm combination wavelengths (*n* = 3). (**f**) Schematic representation of the experimental procedure designed for live cell, real-time OCR measurement ± IRL treatment: Cells underwent 90 min of OGD, followed by 15 min of reoxygenation. (**g**) Control or OGD/R treated cells were placed in a custom light-protected oxygen electrode chamber, and real-time OCR was measured. OCR measurements were monitored before and during illumination with IRL (750 + 950 nm) applied at a power density of 10 mW/cm^2^. (**h**) Quantification from (**g**) of average live cell OCR measurements with and without IRL irradiation in controls and cells subjected to OGD/R (*n* = 3). Representative fluorescent images of mitochondrial superoxide measured using MitoSOX Red at (**i**) 20X magnification and (**j**) 60X magnification. Scale bar, 100 µm and 50 µm, respectively. Quantification of fluorescent intensity of MitoSOX Red normalized to mitochondrial mass using MitoView Green for (**k**) 750 nm, (**l**) 950 nm, and (**m**) 750 + 950 nm combination wavelengths (*n* = 3). Bars represent means ± standard deviation and are presented as normalized to control. ANOVA one-way statistical analysis with Tukey’s post hoc multiple comparisons test was used. Statistical significance was defined as *p* < 0.05, with * indicating *p* < 0.05, ** indicating *p* < 0.01, *** indicating *p* < 0.001, and **** indicating *p* < 0.0001.

The reaction of COX and Cyt*c* is the proposed rate-limiting step of the ETC and thus regulates ΔΨ_m_, and their activation contributes to ΔΨ_m_ hyperpolarization during early reperfusion. To measure relative changes in ΔΨ_m_, the ratio-metric probe JC10 was used, which, as a monomer, emits green fluorescence and, at high ΔΨ_m_, influxes into the mitochondria, causing it to aggregate and emit red fluorescence. Relative changes in ΔΨ_m_ can be quantified by the red-to-green fluorescence ratio. To simulate early reperfusion conditions, cells underwent 90 min of OGD followed by 30 min of reoxygenation with or without IRL (Figure 3a). We evaluated inhibitory IRL wavelengths individually (750 or 950 nm) and in combination (750 + 950 nm). Representative fluorescent images showed increased red fluorescence of JC10 in cells subjected to OGD without IRL treatment, indicating ΔΨ_m_ hyperpolarization. In contrast, cells that received the combination treatment (750 + 950 nm) exhibited fluorescent intensity similar to the control group (Figure 3b). Quantification of red-to-green fluorescence ratio confirmed that cells treated with individual IRL wavelengths or their combination following OGD, resulted in ΔΨ_m_ comparable to control levels (Figure 3c–e). As expected, control cells exposed only to IRL but did not undergo OGD/R, showed a decrease of ΔΨ_m_, mediated by the COX-inhibitory effect under basal conditions.

Next, we measured real-time OCR of live, intact cells. Cells were subjected to 90 min of OGD followed by 15 min of reoxygenation (Figure 3f), allowing time for the substrates (glucose and pyruvate) to be taken up. Cells were then immediately collected by scraping from the dish and placed in a custom light-protected oxygen electrode chamber. The basal OCR was measured following OGD/R (Figure 3g), consistent with the maximal COX-activity measurement of sonicated cells (Figure 2e). Basal OCR was higher in OGD/R-treated cells compared to control cells (Figure 3g). During the same OCR reading, the IRL was turned on and the cells were illuminated at a power density of 10 mW/cm^2^ while simultaneously measuring real-time OCR. We observed a ~50% reduction in OCR upon IRL treatment, an effect that was present in both control and OGD/R treated cells (Figure 3g,h). This result confirms the COX-inhibitory effect of the specific IRL wavelengths used in this study and their ability to reduce COX activity, making them an attractive tool for reducing COX-hyperactivity in pathological conditions like OGD/R.

It is known that mitochondrial ROS production correlates positively with ΔΨ_m_ and that increased ROS is a hallmark of I/R injury [59]. Using MitoSOX Red, a mitochondrial superoxide-specific probe, live cell fluorescent imaging showed an increase in superoxide production in cells subjected to only OGD/R (Figure 3i,j). Quantitative analysis of normalized MitoSOX Red signal to MitoView Green staining (control for mitochondrial mass) showed a ~50% increase in mitochondrial superoxide signal in cells that did not receive IRL. Treatment with IRL during simulated reperfusion significantly lowered superoxide generation across all wavelengths and their combinations (Figure 3k–m). Together, these results show the suitability of the OGD/R cell culture model utilized in this study for investigating I/R injury, as it effectively replicates increased ROS production and alterations in COX activity found in tissues undergoing I/R. In conclusion, the application of inhibitory IRL at the onset of reoxygenation prevents ΔΨ_m_ hyperpolarization, thereby reducing subsequent ROS production.

### 3.3. Inhibitory IRL Prevents Mitochondrial Fragmentation and Oxidative Damage

The individual wavelengths alone were sufficient to provide protection against ΔΨ_m_ hyperpolarization and ROS generation, with greater protection when applied in combination, as monitored in Figure 3a,i,j. To be consistent with our animal studies, we used the combination wavelength in our subsequent experiments.

**Figure 4:**
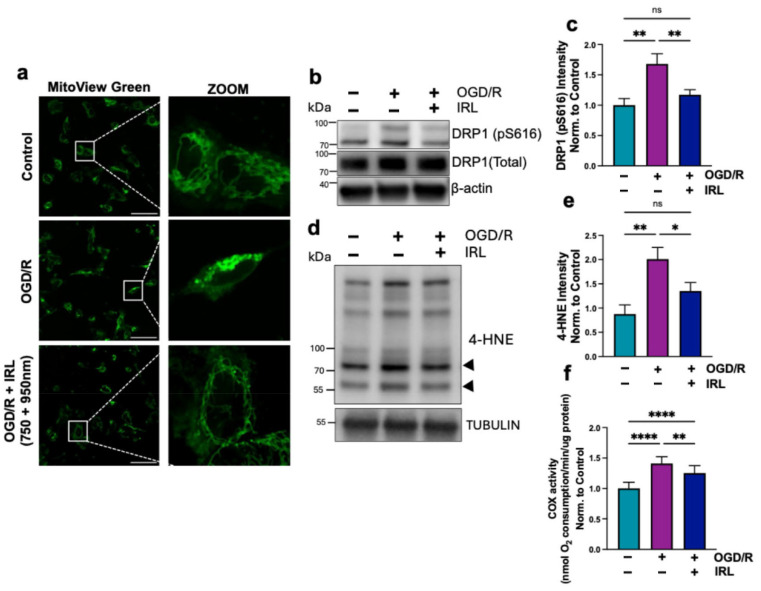
Mitochondrial fragmentation and oxidative damage due to mitochondrial hyperactivity during early reperfusion. (**a**) Mitochondria were stained with MitoView Green after 30 min of reperfusion ± combination IRL. Magnification, 60×; scale bar, 50 µm. (**b**) Western blot of active fission marker DRP1 (pS616), total DRP1, and β-actin to show loading with (**c**) quantification of pS616 DRP1 to total DRP1 (*n* = 3). (**d**) Western blot of lipid peroxidation marker 4-HNE with (**e**) quantification normalized to tubulin (*n* = 3). (**f**) COX activity measurement with whole cells normalized to protein amount (*n* = 3). Bars represent means ± standard deviation and are presented as normalized to control. ANOVA one-way statistical analysis with Tukey’s post hoc multiple comparisons test was used. Statistical significance was defined as *p* < 0.05, with * indicating *p* < 0.05, ** indicating *p* < 0.01, and **** indicating *p* < 0.0001.

Mitochondrial hyper-fragmentation is an indicator of stress and has been reported following I/R [60,61,62]. Mitochondrial staining confirmed cellular responses to OGD/R with increased mitochondrial perinuclear localization and fragmentation, and these morphological changes were effectively prevented by IRL treatment (Figure 4a). This result is supported by Western blot for DRP1, a mitochondrial fission protein, which is activated upon phosphorylation on residue Ser616. Cells undergoing OGD/R without IRL exhibited increased DRP1 phosphorylation relative to total DRP1, a well-established marker for mitophagy and mitochondrial degradation, whereas IRL treatment attenuated this increase in phosphorylated DRP1 (Figure 4b,c).

4-Hydroxynonenal (4-HNE), a lipid peroxidation product and a marker of oxidative damage, was also measured. 4-HNE levels were determined by SDS-PAGE followed by immunoblotting with 4-HNE-specific antibody. Consistent with the increased superoxide production observed using MitoSOX in live-cell imaging (Figure 3h–j), 4-HNE levels in cells that underwent OGD/R without IRL were elevated. IRL application significantly decreased 4-HNE levels (Figure 4d,e). We next measured COX activity after 30 min of reoxygenation to assess whether the COX activity remained elevated. Cells were treated with and without IRL during reoxygenation, collected, and COX activity was measured. There was increased COX activity in both treated and untreated cells that underwent OGD/R (Figure 4f). This result suggests that during early reperfusion (30 min), regulatory phosphorylations are still lost, causing COX to become hyperactive. This also aligns with the ΔΨ_m_ measurements, confirming that inhibitory IRL lowers COX hyperactivity, maintaining activity similar to control levels.

### 3.4. A 2 h Inhibitory IRL Treatment Allows Restoration of COX Activity Sooner

**Figure 5:**
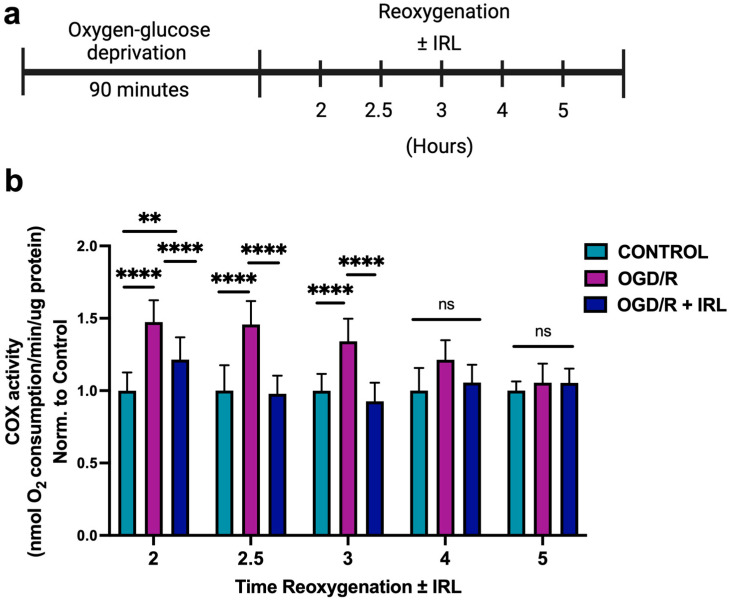
COX activity returns to control levels sooner with IRL treatment. (**a**) Following 90 min of OGD, cells underwent reoxygenation for the indicated times (2, 2.5, 3, 4, 5 h) ± IRL (750 + 950 nm). (**b**) COX activity measurement using whole cells normalized to protein amount (*n* = 3). Bars represent means ± standard deviation and are presented as normalized to control. ANOVA one-way statistical analysis with Tukey’s post hoc multiple comparisons test was used. Statistical significance was defined as *p* < 0.05, with ** indicating *p* < 0.01, and **** indicating *p* < 0.0001.

Our results indicate that COX-inhibitory IRL effectively prevents damage during early reperfusion when applied at the onset of reoxygenation. Previously, we found that 2 h of IRL treatment provided neuroprotection in animal studies [55,56,57]. To evaluate later stages of reperfusion and more prolonged treatment durations, we assessed the time required for COX activity to return to control levels, which would suggest restoration of regulatory phosphorylations. Similarly to the experiments presented in Figure 3 and Figure 4, cells underwent 90 min of OGD. This time, we increased and evaluated different durations of reoxygenation with or without IRL (2, 2.5, 3, 4, and 5 h; Figure 5a). Interestingly, after 2 h of reoxygenation, OCR was still increased in both treated and non-treated, but less in the IRL treated group (Figure 5b). Within 2.5 h of IRL treatment, COX activity had returned to control levels. In contrast, untreated OGD/R still exhibited significantly increased COX activity. Without IRL treatment, COX activity did not return to control levels until 4 h of reoxygenation. These results suggest that a duration of 2 to 2.5 h of IRL treatment is sufficient for mitochondria to return to a normal physiological state, and that regulatory phosphorylations return sooner than without IRL treatment. COX activity remained heightened in untreated cells, suggesting persistent ΔΨ_m_ hyperpolarization and extended ROS production, which would cause additional cellular damage. These results support the justification for a treatment duration of at least 2 h to provide maximal neuroprotection.

### 3.5. Inhibitory IRL Treatment Decreases Cell Death

**Figure 6:**
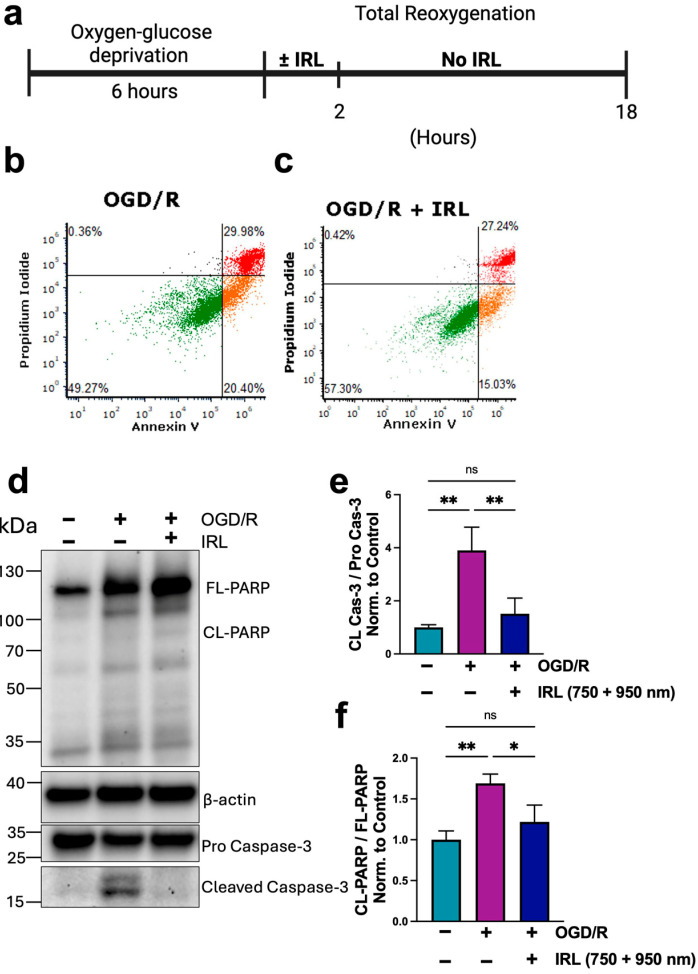
Inhibitory IRL treatment prevents further cell death following late reoxygenation. (**a**) Following 6 h of OGD, cells were treated ± IRL (750 + 950 nm) for 2 h, and then placed in an incubator without IRL for the remaining 16 h, totaling 18 h of reoxygenation. (**b**,**c**) Cell death was quantified using Annexin V/propidium iodide (PI) staining, followed by FACS analysis. Annexin V-positive cells are early apoptotic (orange, right–lower quadrant), PI-positive cells are necrotic (black, upper–left quadrant), and Annexin V/PI positive cells are apoptotic (red, upper–right quadrant). (**d**) Western blot analysis of apoptosis markers, including pro-caspase-3, cleaved (CL) caspase-3, and PARP, and their quantification. β-actin to show loading. (**e**) CL cas-3 to FL cas-3 and (**f**) CL-PARP to FL-PARP (*n* = 3). Bars represent means ± standard deviation and are presented as normalized to control. One-way ANOVA statistical analysis with Tukey’s post hoc multiple comparisons test was used. Statistical significance was defined as *p* < 0.05, with * indicating *p* < 0.05 and ** indicating *p* < 0.01.

We next wanted to evaluate cell death. To note, when 90 min of OGD was used, the level of cell death was minimal in SH-5Y5 cells. This is why we increased the OGD time to push the SH-5Y5 cells to undergo apoptosis and evaluated the effectiveness of the IRL treatment to limit cell death using 6 h of OGD, followed by 2 h of reoxygenation ± IRL, followed by another 16 h of reoxygenation interval to allow execution of the apoptotic signal cascade (Figure 6a). Cells were then collected and stained with Annexin V/propidium iodide (PI) followed by FACS analysis. IRL treated cells had decreased cell death compared to OGD/R alone (Figure 6a–c). Notably, the amount of cell death in the IRL treated group, while lower than without treatment, was still high. Therefore, we evaluated additional markers indicating that cells are continuing to commit to apoptosis. To further support the FACS data, Western blot of apoptosis markers poly (ADP-ribose) polymerase (PARP) and cleaved caspase-3 was performed (Figure 6d). PARP is an enzyme involved in DNA repair activated by several cellular stresses, including hypoxia [63,64]. It is cleaved by caspases to initiate apoptosis, and when overactive, can promote additional cell death. There was increased full-length PARP in both the treated and untreated cells, but there was increased cleaved PARP in the OGD/R alone group when the ratio of cleaved/full-length was quantified (Figure 6f). Additionally, in cells that received IRL treatment, there was significantly decreased cleaved caspase-3 compared to OGD/R alone (Figure 6e). Together, these results show that COX-inhibitory IRL treatment limits cell death.

## 4. Discussion

Blockage of blood flow to the brain results in considerable tissue damage, and the primary method to prevent neurological damage is prompt restoration of blood flow. However, restoration of blood flow causes additional damage to neurons through reperfusion injury, contributing to neurocognitive deficits. Mitochondrial hyperactivity has been known to play a critical role in I/R injury through increased ROS. The general induction of apoptosis following I/R and the connection to mitochondria is proposed as follows: (1) calcium release and sequestration in mitochondria during hypoxia leading to dephosphorylation and activation of mitochondrial proteins including Cyt*c* and COX; (2) ΔΨ_m_ hyperpolarization during early reperfusion due to loss of Cyt*c*/COX regulation; (3) mitochondrial ROS bursts which subsequently initiate apoptosis.

ΔΨ_m_ is generated by the ETC, which couples electron transfer to proton pumping to generate a proton gradient, which is harnessed by ATP synthase (complex V) to generate ATP. Under physiological conditions, this process is highly regulated. COX itself does not generate ROS; however, it has a major role in maintaining optimal intermediate ΔΨ_m_ levels to prevent ROS formation under physiological conditions. The physiological range of ΔΨ_m_ is in the optimal intermediate range of 80–140 mV, which maximizes ATP production and minimizes ROS [64,65,66,67]. Above this range, ATP production cannot be further increased, but rather ROS production increases exponentially. High ΔΨ_m_, above the intermediate range, has been reported in isolated mitochondria studies [68,69]. We and others have proposed that the reason for these discrepancies is due to the differences in intact versus isolated mitochondria. In isolated mitochondria, there is a lack of auxiliary components that contribute to cell signaling cascades and therefore regulation, in addition to the change in phosphorylation state of the ETC complexes. Using mitochondrial isolation methods that preserve protein phosphorylations, our lab has shown that both Cyt*c* and COX are reversibly phosphorylated.

Cellular stress, including ischemia, can lead to signaling cascades that cause dephosphorylations of the ETC complexes and Cyt*c*. During ischemia, there is an influx of Ca^2+^ into the mitochondria, which subsequently activates phosphatases. The result is dephosphorylation of mitochondrial proteins, including important regulatory PTMs of the ETC. We previously found that following cerebral ischemia, Cyt*c* was dephosphorylated, which increased its activity in the reaction with COX [20,31]. Additionally, COX activity was increased in ischemic brain tissue [55]. Due to the difficulty in mapping phosphorylations especially on the hydrophobic catalytic core subunits of COX and the likelihood of multiple phosphorylation sites, there are limitations to characterize phosphorylation state of COX. This is especially true in a cell culture system where material is limited. We present in a relevant cell culture model, a general decrease in mitochondrial protein phosphorylation accompanied by increased COX activity following ischemia (Figure 2c–e). COX-specific activity was measured using whole cells that were collected immediately following the treatment and frozen before analysis. Therefore, measurements were not performed in intact mitochondria and instead used detergent-solubilized COX, which is a direct measurement of COX activity in the absence of a mitochondrial membrane potential. Direct analysis of COX-specific activity has the advantage in that it limits the possible loss (or gain) of post-translational modifications during the time required for mitochondria isolation.

Due to the importance of these regulatory phosphorylations to regulate the ETC and, more specifically, COX and Cyt*c* activity, the loss of these regulations would result in mitochondrial hyperactivity. We propose that COX, as the rate-limiting step of the ETC under physiological conditions in mammals, regulates ΔΨ_m_. With heightened COX activity, there is an increase of ΔΨ_m_ during early reperfusion (Figure 3a–e). Despite undergoing OGD/R, the IRL treated cells, either using the individual wavelengths or their combination, have lowered ΔΨ_m_ levels similar to controls. As a positive control, and to show inhibitory IRL wavelengths can indeed lower ΔΨ_m_ through inhibition of COX, we included cells that did not undergo OGD/R but were treated with IRL alone. These cells show, when compared to controls, there is a statistically significant 25–30% decrease in JC-10 fluorescence, indicating a reduction of ΔΨ_m_. ROS production is positively correlated with ΔΨ_m_ and increases exponentially when exceeding the physiological range. We observed similar findings in live intact cells with increased OCR following OGD/R (Figure 3g,h). Simultaneous OCR measurements with IRL showed a significant reduction in respiration, further supporting the COX-inhibitory effects mediated by these specific wavelengths. We analyzed mitochondrial superoxide production following OGD/R, which was lowered with IRL treatment, with the most protection given by the combination treatment with both wavelengths (Figure 3f–j). There was also increased 4-HNE signal, an indicator of lipid peroxidation due to increased ROS (Figure 4d,e). Supporting the result above, where ROS production was suppressed, the IRL-treated cells did not show significantly increased 4-HNE levels. Cyt*c* is tethered to the inner mitochondrial membrane by cardiolipin. Oxidation of cardiolipin and generation of 4-HNE have been shown to occur when it is exposed to mitochondrial ROS, promoting Cyt*c* release from the mitochondrial intermembrane space into the cytosol to initiate apoptosis [70].

We were also interested to see how long it takes during reperfusion to restore COX activity to basal levels in the presence and absence of IRL treatment. This information may be clinically useful as it can guide treatment duration decisions. A previous report suggested that COX activity decreases at later stages of reperfusion, starting to decline within the first hour of reperfusion with maximal inhibition by 24 h [71]. Indeed, in both the untreated and treated cells that underwent OGD/R, COX activity was still significantly increased after 30 min of reoxygenation (Figure 4f). These results indicate that COX has not regained its regulatory phosphorylations and would still be hyperactive if not for the inhibitory IRL. Interestingly, at 2 h of simulated reperfusion, untreated cells still had a 50% increase in COX activity compared to controls, while IRL treated cells showed 25–30% increased COX activity from control, suggesting that they recover faster (Figure 5a,b). Therefore, we propose that after 2 h of IRL treatment during reperfusion, a fraction of the cells have regained regulatory status to COX. At 2.5 h of treatment, OCR had returned to control levels, unlike untreated cells, which still remained high. In the untreated cells, it took 4–5 h for COX activity to normalize. These results indicate that without IRL treatment, mitochondria in those cells that survive would be hyperactive up to 4–5 h after reoxygenation and that a delayed treatment might still be useful in the context of brain I/R injury. This concept is supported by or previous work showing that a 30- and 60 min delay of treatment onset into reperfusion still provides robust neuroprotection in a rat model of global brain I/R injury [55]. The amount of ROS produced in the extended time window in the absence of IRL treatment would cause further damage to the mitochondria and the cell. The damage that accumulates during reperfusion without treatment likely augments mitochondrial damage and reduces ETC function, eventually leading to loss of respiration, collapse of ΔΨ_m_, and further cell death. This is consistent with our data showing increased apoptosis in the untreated cells and previous reports showing loss of COX activity and ΔΨ_m_ at late-stage reperfusion [72,73,74].

Some pharmacological attempts to prevent mitochondrial hyperactivity have directly targeted ΔΨ_m_ hyperpolarization. Proton ionophores are mitochondrial uncouplers which allow proton leak across the IMM, dissipating ΔΨ_m_, and have shown to prevent ΔΨ_m_ hyperpolarization and prevented ROS [39]. In small concentrations, uncouplers can provide protection, whereas at higher concentrations, they can further exacerbate mitochondrial dysfunction [75]. However, these results support our model of I/R injury and that targeting ΔΨ_m_ provides neuroprotection. Nevertheless, the use of these compounds poses a therapeutic barrier due to the small therapeutic window and because they act systemically, and an overdose could lead to respiratory collapse in addition to the inherent difficulty to deliver drugs to mitochondria at the time of reperfusion because they rely on blood flow. There has been interest in other approaches to target COX, such as the use of gasotransmitters including carbon monoxide (CO), nitric oxide (NO), and hydrogen sulfide (H_2_S), which can freely cross membranes and do not fully rely on blood flow [76]. However, these molecules have a short half-life and, as above, potential overdosing may induce further damage.

## 5. Conclusions

**Figure 7:**
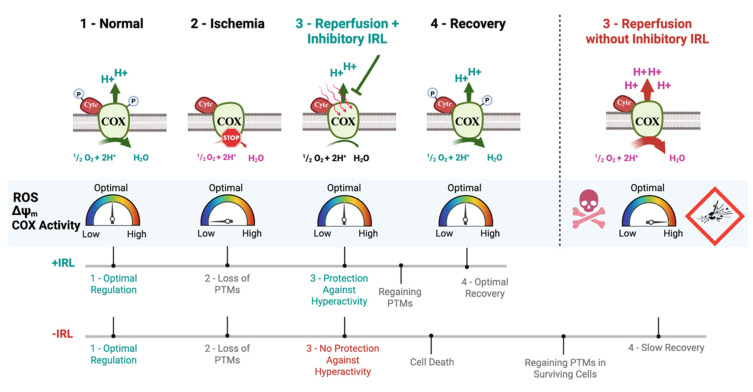
Model of protection I/R injury with inhibitory IRL. Treatment with IRL during early reperfusion prevents ΔΨ_m_ hyperpolarization and ROS, allowing optimal cellular recovery and prevention of further cell death.

We have shown that inhibitory IRL applied at the time of reoxygenation can maintain ΔΨ_m_ near control levels by targeting COX, suppressing mitochondrial superoxide production. In contrast to pharmacological approaches that rely on blood flow and act systemically, IRL, as a therapeutic, is advantageous as it is non-invasive, non-pharmacological, and, therefore, does not rely on blood flow. Its effect is immediate, without delay, and it can be applied locally to the affected organ. This allows for protection against mitochondrial and cellular stress, such as mitochondrial fragmentation and lipid peroxidation, and decreases further cell death (Figure 7). We have also shown that COX activity is still increased up to 4–5 h following reoxygenation in the absence of IRL treatment, indicating mitochondrial hyperactivity is present over an extended time period that can be cut in half with IRL treatment, allowing faster tissue recovery. We previously reported that the 750 and 950 nm wavelengths penetrate 4 cm deep into the brain [54,77], allowing the delivery of therapeutic doses to the target area with robust neuroprotection and prevention of cognitive deficits in both small and large animal models [55,56,57]. We therefore propose that this could be a realistic and mechanism-based therapy for I/R injury with clear advantages over pharmacological approaches, the latter of which did not translate into the clinic. The cell culture model described here may be useful to simulate acute and chronic neuronal stress conditions, ranging from brain I/R, spinal cord injury, traumatic brain injury, and tinnitus.

## Data Availability

Data are contained within the article.

## Abbreviations

The following abbreviations are used in this manuscript:

COX	Cytochrome *c* oxidase
Cyt*c*	Cytochrome *c*
ROS	Reactive oxygen species
OGD/R	Oxygen–glucose deprivation/reoxygenation
I/R	Ischemia/reperfusion
IRL	Infrared light
ETC	Electron transport chain
PTM	Post-translation modifications
OCR	Oxygen consumption rate

## References

[1] Martin S.S., Aday A.W., Allen N.B., Almarzooq Z.I., Anderson C.A.M., Arora P., Avery C.L., Baker-Smith C.M., Bansal N., Beaton A.Z. (2025). 2025 Heart Disease and Stroke Statistics: A Report of US and Global Data From the American Heart Association. Circulation.

[2] Morse P.T., Wan J., Bell J., Lee I., Goebel D.J., Malek M.H., Sanderson T.H., Hüttemann M. (2022). Sometimes less is more: Inhibitory infrared light during early reperfusion calms hyperactive mitochondria and suppresses reperfusion injury. Biochem. Soc. Trans..

[3] St-Pierre J., Buckingham J.A., Roebuck S.J., Brand M.D. (2002). Topology of superoxide production from different sites in the mitochondrial electron transport chain. J. Biol. Chem..

[4] Liu S.S. (1999). Cooperation of a “reactive oxygen cycle” with the Q cycle and the proton cycle in the respiratory chain--superoxide generating and cycling mechanisms in mitochondria. J. Bioenerg. Biomembr..

[5] Starkov A.A., Fiskum G. (2003). Regulation of brain mitochondrial H2O2 production by membrane potential and NAD(P)H redox state. J. Neurochem..

[6] Liu S.S. (2010). Mitochondrial Q cycle-derived superoxide and chemiosmotic bioenergetics. Ann. N. Y. Acad. Sci..

[7] Korshunov S.S., Skulachev V.P., Starkov A.A. (1997). High protonic potential actuates a mechanism of production of reactive oxygen species in mitochondria. FEBS Lett..

[8] Rottenberg H., Covian R., Trumpower B.L. (2009). Membrane potential greatly enhances superoxide generation by the cytochrome bc1 complex reconstituted into phospholipid vesicles. J. Biol. Chem..

[9] Kaim G., Dimroth P. (1999). ATP synthesis by F-type ATP synthase is obligatorily dependent on the transmembrane voltage. EMBO J..

[10] Dalmonte M.E., Forte E., Genova M.L., Giuffre A., Sarti P., Lenaz G. (2009). Control of respiration by cytochrome c oxidase in intact cells: Role of the membrane potential. J. Biol. Chem..

[11] Gnaiger E., Lassnig B., Kuznetsov A., Rieger G., Margreiter R. (1998). Mitochondrial oxygen affinity, respiratory flux control and excess capacity of cytochrome c oxidase. J. Exp. Biol..

[12] Kunz W.S., Kudin A., Vielhaber S., Elger C.E., Attardi G., Villani G. (2000). Flux control of cytochrome c oxidase in human skeletal muscle. J. Biol. Chem..

[13] Piccoli C., Scrima R., Boffoli D., Capitanio N. (2006). Control by cytochrome c oxidase of the cellular oxidative phosphorylation system depends on the mitochondrial energy state. Biochem. J..

[14] Villani G., Attardi G. (1997). In vivo control of respiration by cytochrome c oxidase in wild-type and mitochondrial DNA mutation-carrying human cells. Proc. Natl. Acad. Sci. USA.

[15] Wiedemann F.R., Kunz W.S. (1998). Oxygen dependence of flux control of cytochrome c oxidase—Implications for mitochondrial diseases. FEBS Lett..

[16] Pham L., Arroum T., Wan J., Pavelich L., Bell J., Morse P.T., Lee I., Grossman L.I., Sanderson T.H., Malek M.H. (2024). Regulation of mitochondrial oxidative phosphorylation through tight control of cytochrome c oxidase in health and disease—Implications for ischemia/reperfusion injury, inflammatory diseases, diabetes, and cancer. Redox Biol..

[17] Lee I., Salomon A.R., Ficarro S., Mathes I., Lottspeich F., Grossman L.I., Hüttemann M. (2005). cAMP-dependent tyrosine phosphorylation of subunit I inhibits cytochrome c oxidase activity. J. Biol. Chem..

[18] Lee I., Salomon A.R., Yu K., Doan J.W., Grossman L.I., Hüttemann M. (2006). New prospects for an old enzyme: Mammalian cytochrome c is tyrosine-phosphorylated in vivo. Biochemistry.

[19] Yu H., Lee I., Salomon A.R., Yu K., Hüttemann M. (2008). Mammalian liver cytochrome c is tyrosine-48 phosphorylated in vivo, inhibiting mitochondrial respiration. Biochim. Biophys. Acta.

[20] Sanderson T.H., Mahapatra G., Pecina P., Ji Q., Yu K., Sinkler C., Varughese A., Kumar R., Bukowski M.J., Tousignant R.N. (2013). Cytochrome C is tyrosine 97 phosphorylated by neuroprotective insulin treatment. PLoS ONE.

[21] Prabu S.K., Anandatheerthavarada H.K., Raza H., Srinivasan S., Spear J.F., Avadhani N.G. (2006). Protein kinase A-mediated phosphorylation modulates cytochrome c oxidase function and augments hypoxia and myocardial ischemia-related injury. J. Biol. Chem..

[22] Zaidan E., Sims N.R. (1994). The calcium content of mitochondria from brain subregions following short-term forebrain ischemia and recirculation in the rat. J. Neurochem..

[23] Kristian T., Pivovarova N.B., Fiskum G., Andrews S.B. (2007). Calcium-induced precipitate formation in brain mitochondria: Composition, calcium capacity, and retention. J. Neurochem..

[24] Puka-Sundvall M., Gajkowska B., Cholewinski M., Blomgren K., Lazarewicz J.W., Hagberg H. (2000). Subcellular distribution of calcium and ultrastructural changes after cerebral hypoxia-ischemia in immature rats. Brain Res. Dev. Brain Res..

[25] Balaban R.S. (2002). Cardiac energy metabolism homeostasis: Role of cytosolic calcium. J. Mol. Cell Cardiol..

[26] Robb-Gaspers L.D., Burnett P., Rutter G.A., Denton R.M., Rizzuto R., Thomas A.P. (1998). Integrating cytosolic calcium signals into mitochondrial metabolic responses. EMBO J..

[27] Ankarcrona M., Dypbukt J.M., Orrenius S., Nicotera P. (1996). Calcineurin and mitochondrial function in glutamate-induced neuronal cell death. FEBS Lett..

[28] Hopper R.K., Carroll S., Aponte A.M., Johnson D.T., French S., Shen R.F., Witzmann F.A., Harris R.A., Balaban R.S. (2006). Mitochondrial matrix phosphoproteome: Effect of extra mitochondrial calcium. Biochemistry.

[29] Bender E., Kadenbach B. (2000). The allosteric ATP-inhibition of cytochrome c oxidase activity is reversibly switched on by cAMP-dependent phosphorylation. FEBS Lett..

[30] Lee I., Bender E., Kadenbach B. (2002). Control of mitochondrial membrane potential and ROS formation by reversible phosphorylation of cytochrome c oxidase. Mol. Cell. Biochem..

[31] Kalpage H.A., Vaishnav A., Liu J., Varughese A., Wan J., Turner A.A., Ji Q., Zurek M.P., Kapralov A.A., Kagan V.E. (2019). Serine-47 phosphorylation of cytochrome c in the mammalian brain regulates cytochrome c oxidase and caspase-3 activity. FASEB J..

[32] Iijima T., Mishima T., Akagawa K., Iwao Y. (2006). Neuroprotective effect of propofol on necrosis and apoptosis following oxygen-glucose deprivation--relationship between mitochondrial membrane potential and mode of death. Brain Res..

[33] Iijima T., Mishima T., Akagawa K., Iwao Y. (2003). Mitochondrial hyperpolarization after transient oxygen-glucose deprivation and subsequent apoptosis in cultured rat hippocampal neurons. Brain Res..

[34] Zhang Y., Marcillat O., Giulivi C., Ernster L., Davies K.J. (1990). The oxidative inactivation of mitochondrial electron transport chain components and ATPase. J. Biol. Chem..

[35] Kushnareva Y., Murphy A.N., Andreyev A. (2002). Complex I-mediated reactive oxygen species generation: Modulation by cytochrome c and NAD(P)+ oxidation-reduction state. Biochem. J..

[36] Kunimatsu T., Kobayashi K., Yamashita A., Yamamoto T., Lee M.C. (2011). Cerebral reactive oxygen species assessed by electron spin resonance spectroscopy in the initial stage of ischemia-reperfusion are not associated with hypothermic neuroprotection. J. Clin. Neurosci..

[37] Liu R.R., Murphy T.H. (2009). Reversible cyclosporin A-sensitive mitochondrial depolarization occurs within minutes of stroke onset in mouse somatosensory cortex in vivo: A two-photon imaging study. J. Biol. Chem..

[38] Pandya J.D., Pauly J.R., Sullivan P.G. (2009). The optimal dosage and window of opportunity to maintain mitochondrial homeostasis following traumatic brain injury using the uncoupler FCCP. Exp. Neurol..

[39] Brennan J.P., Southworth R., Medina R.A., Davidson S.M., Duchen M.R., Shattock M.J. (2006). Mitochondrial uncoupling, with low concentration FCCP, induces ROS-dependent cardioprotection independent of KATP channel activation. Cardiovasc. Res..

[40] Choi K., Kim J., Kim G.W., Choi C. (2009). Oxidative stress-induced necrotic cell death via mitochondira-dependent burst of reactive oxygen species. Curr. Neurovasc. Res..

[41] Chan P.H., Kawase M., Murakami K., Chen S.F., Li Y., Calagui B., Reola L., Carlson E., Epstein C.J. (1998). Overexpression of SOD1 in transgenic rats protects vulnerable neurons against ischemic damage after global cerebral ischemia and reperfusion. J. Neurosci..

[42] Murakami K., Kondo T., Kawase M., Li Y., Sato S., Chen S.F., Chan P.H. (1998). Mitochondrial susceptibility to oxidative stress exacerbates cerebral infarction that follows permanent focal cerebral ischemia in mutant mice with manganese superoxide dismutase deficiency. J. Neurosci..

[43] Fujimura M., Morita-Fujimura Y., Noshita N., Sugawara T., Kawase M., Chan P.H. (2000). The cytosolic antioxidant copper/zinc-superoxide dismutase prevents the early release of mitochondrial cytochrome c in ischemic brain after transient focal cerebral ischemia in mice. J. Neurosci..

[44] Christophe M., Nicolas S. (2006). Mitochondria: A target for neuroprotective interventions in cerebral ischemia-reperfusion. Curr. Pharm. Des..

[45] Niizuma K., Endo H., Chan P.H. (2009). Oxidative stress and mitochondrial dysfunction as determinants of ischemic neuronal death and survival. J. Neurochem..

[46] Weigl M., Tenze G., Steinlechner B., Skhirtladze K., Reining G., Bernardo M., Pedicelli E., Dworschak M. (2005). A systematic review of currently available pharmacological neuroprotective agents as a sole intervention before anticipated or induced cardiac arrest. Resuscitation.

[47] Cemeli E., Baumgartner A., Anderson D. (2009). Antioxidants and the Comet assay. Mutat. Res..

[48] Hazeki O., Tamura M. (1988). Quantitative analysis of hemoglobin oxygenation state of rat brain in situ by near-infrared spectrophotometry. J. Appl. Physiol..

[49] Karu T.I., Afanas’eva N.I. (1995). Cytochrome c oxidase as the primary photoacceptor upon laser exposure of cultured cells to visible and near IR-range light. Dokl. Akad. Nauk..

[50] Mason M.G., Nicholls P., Cooper C.E. (2014). Re-evaluation of the near infrared spectra of mitochondrial cytochrome c oxidase: Implications for non invasive in vivo monitoring of tissues. Biochim. Biophys. Acta.

[51] Wharton D.C., Tzagoloff A. (1964). Studies on the Electron Transfer System. Lvii. The near Infrared Absorption Band of Cytochrome Oxidase. J. Biol. Chem..

[52] Wong-Riley M.T., Liang H.L., Eells J.T., Chance B., Henry M.M., Buchmann E., Kane M., Whelan H.T. (2005). Photobiomodulation directly benefits primary neurons functionally inactivated by toxins: Role of cytochrome c oxidase. J. Biol. Chem..

[53] Eells J.T., Wong-Riley M.T., VerHoeve J., Henry M., Buchman E.V., Kane M.P., Gould L.J., Das R., Jett M., Hodgson B.D. (2004). Mitochondrial signal transduction in accelerated wound and retinal healing by near-infrared light therapy. Mitochondrion.

[54] Morse P.T., Goebel D.J., Wan J., Tuck S., Hakim L., Hüttemann C.L., Malek M.H., Lee I., Sanderson T.H., Hüttemann M. (2021). Cytochrome c oxidase-modulatory near-infrared light penetration into the human brain: Implications for the noninvasive treatment of ischemia/reperfusion injury. IUBMB Life.

[55] Sanderson T.H., Wider J.M., Lee I., Reynolds C.A., Liu J., Lepore B., Tousignant R., Bukowski M.J., Johnston H., Fite A. (2018). Inhibitory modulation of cytochrome c oxidase activity with specific near-infrared light wavelengths attenuates brain ischemia/reperfusion injury. Sci. Rep..

[56] Wider J.M., Gruley E., Morse P.T., Wan J., Lee I., Anzell A.R., Fogo G.M., Mathieu J., Hish G., O’Neil B. (2023). Modulation of mitochondrial function with near-infrared light reduces brain injury in a translational model of cardiac arrest. Crit. Care.

[57] Strubakos C.D., Malik M., Wider J.M., Lee I., Reynolds C.A., Mitsias P., Przyklenk K., Hüttemann M., Sanderson T.H. (2020). Non-invasive treatment with near-infrared light: A novel mechanisms-based strategy that evokes sustained reduction in brain injury after stroke. J. Cereb. Blood Flow. Metab..

[58] Li Y., Ren D. (2021). Two-Dimensional Gel Electrophoresis and Pro-Q Diamond Phosphoprotein Stain-Based Plant Phosphosproteomics. Methods Mol. Biol..

[59] Sanderson T.H., Reynolds C.A., Kumar R., Przyklenk K., Hüttemann M. (2013). Molecular mechanisms of ischemia-reperfusion injury in brain: Pivotal role of the mitochondrial membrane potential in reactive oxygen species generation. Mol. Neurobiol..

[60] Kumar R., Bukowski M.J., Wider J.M., Reynolds C.A., Calo L., Lepore B., Tousignant R., Jones M., Przyklenk K., Sanderson T.H. (2016). Mitochondrial dynamics following global cerebral ischemia. Mol. Cell Neurosci..

[61] Maneechote C., Palee S., Chattipakorn S.C., Chattipakorn N. (2017). Roles of mitochondrial dynamics modulators in cardiac ischaemia/reperfusion injury. J. Cell Mol. Med..

[62] Ong S.B., Subrayan S., Lim S.Y., Yellon D.M., Davidson S.M., Hausenloy D.J. (2010). Inhibiting mitochondrial fission protects the heart against ischemia/reperfusion injury. Circulation.

[63] Liu S., Luo W., Wang Y. (2022). Emerging role of PARP-1 and PARthanatos in ischemic stroke. J. Neurochem..

[64] Chen Y.L., Wang Y., Fang Q.Y., Wang T., Chen C., Gao T.Y., Wu M., Zhang W.P., Lu Y.B. (2024). PARP-1 inhibitor alleviates cerebral ischemia/reperfusion injury by reducing PARylation of HK-1 and LDH in mice. Eur. J. Pharmacol..

[65] Wan B., Doumen C., Duszynski J., Salama G., Vary T.C., LaNoue K.F. (1993). Effects of cardiac work on electrical potential gradient across mitochondrial membrane in perfused rat hearts. Am. J. Physiol..

[66] Zhang H., Huang H.M., Carson R.C., Mahmood J., Thomas H.M., Gibson G.E. (2001). Assessment of membrane potentials of mitochondrial populations in living cells. Anal. Biochem..

[67] Backus M., Piwnica-Worms D., Hockett D., Kronauge J., Lieberman M., Ingram P., LeFurgey A. (1993). Microprobe analysis of Tc-MIBI in heart cells: Calculation of mitochondrial membrane potential. Am. J. Physiol..

[68] Nicholls D.G. (1974). The influence of respiration and ATP hydrolysis on the proton-electrochemical gradient across the inner membrane of rat-liver mitochondria as determined by ion distribution. Eur. J. Biochem..

[69] Labajova A., Vojtiskova A., Krivakova P., Kofranek J., Drahota Z., Houstek J. (2006). Evaluation of mitochondrial membrane potential using a computerized device with a tetraphenylphosphonium-selective electrode. Anal. Biochem..

[70] Ott M., Robertson J.D., Gogvadze V., Zhivotovsky B., Orrenius S. (2002). Cytochrome c release from mitochondria proceeds by a two-step process. Proc. Natl. Acad. Sci. USA.

[71] Racay P., Tatarkova Z., Chomova M., Hatok J., Kaplan P., Dobrota D. (2009). Mitochondrial calcium transport and mitochondrial dysfunction after global brain ischemia in rat hippocampus. Neurochem. Res..

[72] Li F., Li D., Tang S., Liu J., Yan J., Chen H., Yan X. (2021). Quercetin Protects H9c2 Cardiomyocytes against Oxygen-Glucose Deprivation/Reoxygenation-Induced Oxidative Stress and Mitochondrial Apoptosis by Regulating the ERK1/2/DRP1 Signaling Pathway. Evid. Based Complement. Altern. Med..

[73] Ye M., Wu H., Li S. (2021). Resveratrol alleviates oxygen/glucose deprivation/reoxygenation-induced neuronal damage through induction of mitophagy. Mol. Med. Rep..

[74] Zhou T., Mo J., Xu W., Hu Q., Liu H., Fu Y., Jiang J. (2023). Mild hypothermia alleviates oxygen-glucose deprivation/reperfusion-induced apoptosis by inhibiting ROS generation, improving mitochondrial dysfunction and regulating DNA damage repair pathway in PC12 cells. Apoptosis.

[75] Han Y.H., Kim S.H., Kim S.Z., Park W.H. (2009). Carbonyl cyanide p-(trifluoromethoxy) phenylhydrazone (FCCP) as an O2(*-) generator induces apoptosis via the depletion of intracellular GSH contents in Calu-6 cells. Lung Cancer.

[76] Andreadou I., Iliodromitis E.K., Rassaf T., Schulz R., Papapetropoulos A., Ferdinandy P. (2015). The role of gasotransmitters NO, H2S and CO in myocardial ischaemia/reperfusion injury and cardioprotection by preconditioning, postconditioning and remote conditioning. Br. J. Pharmacol..

[77] Morse P.T., Tuck S., Kerns M., Goebel D.J., Wan J., Waddell T., Wider J.M., Hüttemann C.L., Malek M.H., Lee I. (2023). Non-invasive treatment of ischemia/reperfusion injury: Effective transmission of therapeutic near-infrared light into the human brain through soft skin-conforming silicone waveguides. Bioeng. Transl. Med..

